# The Moderating Role of Digital Literacy in the Relationship Between Cognitive Function and Instrumental Activities of Daily Living Among Community-Dwelling Older Adults

**DOI:** 10.3390/healthcare14131918

**Published:** 2026-07-01

**Authors:** Taejeong Jang

**Affiliations:** Department of Nursing, Joongbu University, Geumsan-gun 32713, Republic of Korea; tjjang@joongbu.ac.kr

**Keywords:** older adults, cognitive function, activities of daily life, depression, digital literacy

## Abstract

**Background**: As population aging accelerates, maintaining functional independence among older adults has become a critical public health priority. Cognitive function is a well-established determinant of instrumental activities of daily living (IADL); however, the role of digital literacy in shaping this relationship remains unclear. In the context of increasing digitalization, digital literacy may serve as a key factor supporting aging in place. Therefore, this study aimed to examine the moderating effect of digital literacy on the relationship between cognitive function and IADL among community-dwelling older adults. **Methods**: This descriptive cross-sectional study was conducted among 147 community-dwelling older adults aged 75 years and older. Cognitive function was assessed using the Korean version of the Montreal Cognitive Assessment (MoCA-K), IADL was measured using the Korean Instrumental Activities of Daily Living (K-IADL) scale, and digital literacy was evaluated using an 11-item dichotomous scale. Data were analyzed using descriptive statistics, Pearson’s correlation analysis, and hierarchical regression analysis. The moderating effect of digital literacy was tested using the PROCESS macro. **Results**: Cognitive function was positively correlated with digital literacy (r = 0.56, *p* < 0.001) and IADL (r = 0.26, *p* < 0.001), and digital literacy was also positively correlated with IADL (r = 0.25, *p* < 0.001). In the regression analysis, cognitive function significantly predicted IADL (B = 0.11, *p* = 0.004), but this effect became non-significant after including digital literacy in the model. A significant moderating effect of digital literacy was observed (B = −0.03, *p* < 0.001). Conditional effects analysis revealed that cognitive function significantly influenced IADL only among older adults with low levels of digital literacy (B = 0.12, *p* = 0.005), whereas no significant effect was found at moderate or high levels of digital literacy. **Conclusions**: Digital literacy significantly moderates the relationship between cognitive function and ADL among community-dwelling older adults. These findings suggest that digital literacy may function as a compensatory mechanism that mitigates the impact of cognitive decline on daily functioning. Enhancing digital literacy may represent an effective strategy to promote functional independence and support aging in place in an increasingly digital society.

## 1. Introduction

### 1.1. Background

The aging population is a worldwide phenomenon. As a result, the population aged ≥75 years is growing rapidly. One of the main goals of healthcare and public policy is to make sure older people can continue to live safely and independently in their communities, not only from the point of view of the distribution of resources but also for social stability [1]. The ability to perform daily tasks, both basic (ADL) and instrumental (IADL), is a primary component of aging well and enjoying a high quality of life for those decisions made by older people living in the community [2].

Declines in cognitive areas, especially memory and executive functions, make it very difficult for older adults to carry out instrumental activities like taking medication and handling finances [3,4]. Moreover, the link between cognitive function and IADL is not straightforward [5] because the connection between them is often mediated by environmental or other factors. Low cognitive function still stands as the main cause of the loss of individual independence, early nursing home placement, and increased healthcare expenses [5]. However, the relationship between cognitive function and IADL is not linear, and research shows that contextual and environmental factors are likely to play a role in that association.

Driven by rapid digital transformation, the recent use of digital devices has greatly increased the utility and convenience of our everyday lives and healthcare. Digital technologies are increasingly considered for obtaining health information, telemedicine, social communication, and management of daily activities. In this context, digital literacy, which can be defined as the ability of a person to understand, access, evaluate, and appropriately use digital information, represents a critical determinant of health equity, an important factor in healthcare disparities and overall health status [6]. Prior research shows that digital literacy of older adults is positively related to better health management, more frequent health-promoting behavior, and more social integration [7,8].

The digitally literate older generation can benefit from using assistive technologies and online health portals through which they can independently maintain their community dwellings [9]. Moreover, those with low digital engagement may become more vulnerable to health disparities in the context of increasingly digitalized health systems [10]. Additionally, digital literacy is a resource that can overcome the functional limitations that result from cognitive decline [9]. Theoretically, this relationship can be understood through the Selection, Optimization, and Compensation (SOC) framework, which posits that older adults utilize external resources to compensate for age-related biological losses. In a highly digitalized society, digital literacy functions as a technological compensatory mechanism—an extension of cognitive reserve—that allows older adults to optimize their remaining capacities. By utilizing digital proxies, individuals can bypass traditional cognitive barriers to complete complex daily tasks. For instance, through the use of digital proxies, such as smart notifications and navigational aids, seniors can remain independent while their cognitive capacities are reduced.

However, limited research has been conducted on how digital literacy might be a moderator in the relationship between cognitive function and IADL tasks, especially among the population aged ≥75 years [6,7]. Understanding this relationship is essential for developing policies and clinical approaches that encourage both digital inclusion and cognitive health. In line with this, the present study examines whether digital literacy is a moderator variable in the link between cognitive function and daily life performance in older people. In particular, this research sheds light on the synergy between specific aspects of cognitive function and digital literacy, thereby supporting lifelong performance abilities in the current society.

### 1.2. Aims and Objectives

This study investigated the moderating capacity of digital literacy regarding the impact of cognitive function on IADL among community-dwelling older adults. The specific objectives of this study were to: (1) evaluate the levels of cognitive function, digital literacy, and IADL among the participants; (2) examine the correlations among these three primary variables; (3) identify the independent predictors of IADL while controlling for socio-demographic covariates; and (4) verify whether digital literacy moderates the relationship between cognitive function and functional IADL performance.

## 2. Materials and Methods

### 2.1. Study Design

This descriptive research was carried out to verify the moderating role of digital literacy in the link between cognitive functioning and ability for daily life performance of the elderly living in communities from 30 June 2024 through 30 January 2025.

### 2.2. Setting and Sampling

Participants were recruited via convenience sampling from three public senior welfare centers located in D city, South Korea. G*Power 3.1.9.7 software (Heinrich-Heine-University Düsseldorf in Germany) was used to calculate the necessary sample size. Since the main goal of the study was to perform multiple regression analysis to explore how digital literacy may moderate the association between cognitive function and IADL, the power analysis was performed with a significance level of 0.05, statistical power of 0.80, medium effect size of 0.15, and 10 predictors. The lowest sample size was 122. However, allowing for an attrition rate of 20%, mainly due to missing data or cognitive fatigue, the final number for recruitment was set at 150. The inclusion criteria were individuals aged 75 or older who could comprehend the study’s purpose. The exclusion criteria were as follows: (1) individuals hospitalized within the last 3 months; (2) individuals with intellectual disabilities; and (3) individuals with severe communication disorders, specifically defined as an inability to follow simple two-step verbal instructions or severe expressive/receptive aphasia due to prior neurological events that prevents independent survey completion.

### 2.3. Data Collection Tools

The survey assessed six general socio-demographic characteristics, six areas of cognitive function, and eleven questions on digital literacy. General characteristics of participants included their age, gender, education, marital status, income, and comorbidity.

#### 2.3.1. Cognitive Function

Cognitive function was measured using the Korean Montreal Cognitive Assessment Scale (MoCA-K). Originally developed by Nasreddine et al. for the detection of mild cognitive impairment [11], this instrument consists of six areas: visuospatial/executive functions, vocabulary, memory, attention, language, abstraction/delayed recall, and persistence. Those who exhibited a total score below 23 points are considered to have mild cognitive impairment, while scoring on the whole is positively linked to cognitive capacity. The internal consistency of the tool, as indicated by Cronbach’s ⍺, was 0.84 [12] and even better at 0.86 in the present study.

#### 2.3.2. Daily Living Activities

We selected the Korean-style Instrumental Activities of Daily Living (K-IADL) tool [13] to measure complex functional capabilities. The set of 10 items evaluates activities such as grooming, housework, preparing meals, laundry, short-range outings, using transportation, purchasing products, managing finances, using the phone, and taking medication. Scoring options ranged from 0 (completely independent) to 3 (completely dependent), which depended on the level of performance, with higher scores indicating lower abilities to perform daily life activities. For statistical parsimony and co-interpretative consistency, scores were reverse-coded so that higher values reflect superior functional capacity. The internal consistency (Cronbach’s α) was 0.95 in the previous literature [14], while it was 0.93 in the present study.

#### 2.3.3. Digital Literacy

To evaluate the digital literacy of the participants, 11 items were used to cover essential digital tasks that most people perform daily. These included instant messaging, searching the web for information, and digital e-commerce [15]. The tool used by the participants is based on the conceptualization models proposed by Eshet-Alkalai [16]. The tool breaks down digital literacy into three main areas: information retrieval (e.g., ‘Can you search for health information on the web?’), interpersonal communication (e.g., ‘Can you send an instant message?’), and media usage (e.g., ‘Can you use mobile e-commerce?’). Given the nature of the elderly population, for procedural reasons, we used a dichotomous score system (yes = 1, no = 0): the higher the score, the greater the digital capability. Construct validity was established in prior Korean gerontological research, and the tool demonstrated an internal consistency of Cronbach’s α = 0.82 in this study.

#### 2.3.4. Data Collection

Data collection was carried out after participants gave their informed consent by enrolling in the study voluntarily. In terms of the study variables, cognitive testing was performed by research assistants with nursing training. The other survey measures were completed by the participants themselves, though they were monitored by research assistants. It took an average of 20–30 min to finish, and participants were given incentives as a way to thank them for their participation.

#### 2.3.5. Ethical Approval

Before starting the study, we obtained institutional approval from Joongbu University’s institutional review board (Approval No: JBU-2024-IRB-0042). Moreover, a verbal explanation was provided to the participants regarding the objectives and procedures of the study, and we obtained their informed consent in written form. The research was carried out in full compliance with the Declaration of Helsinki.

#### 2.3.6. Data Analysis

The dataset was analyzed with SPSS 27.0 (IBM Corp., Armonk, NY, USA). The normality of distribution for variables was mostly tested through univariate frequency analysis and descriptive statistics. Mean and standard deviation (SD) statistics were computed, while Pearson’s correlation was used to find relationships between variables. To test for a moderation effect of digital literacy, Hayes’ PROCESS software (Andrew F. Hayes, Calgary, AB, Canada) was employed to implement a moderation model (Model 1). In this model, IADL, cognitive function, and digital literacy were defined as dependent, independent, and moderator variables, respectively.

## 3. Results

### 3.1. General Characteristics of Participants

The average age of the participants was 79 years (SD ± 4.33), and 117 (79.6%) were female in Table 1. Educational background consisted primarily of elementary school completion (33.3%), and over half the group (52.4%) were widowed. The economic level was very “low” for 85.7% of the population, and 93.9% suffered from concomitant diseases.

Differences in IADL according to baseline characteristics were only statistically significant for comorbidity status. In particular, those who had musculoskeletal diseases were less functionally independent than those with other illnesses or no disease at all.

Functional capacity did not significantly differ by age, gender, education, marital status, or economic income. Only comorbidity status exhibited a statistically significant relationship with baseline functional independence (F = 6.23, *p* < 0.001). Accordingly, comorbidity status was systematically treated as a baseline covariate in subsequent regression and moderation models to ensure controlled equivalence.

### 3.2. Levels and Correlations of Study Variables

The correlation matrix, including cognitive function, digital literacy, and IADL, is shown in Table 2. Cognitive function was moderately to strongly positively associated with digital literacy (*r* = 0.56) and positively correlated with IADL (*r* = 0.26). Digital literacy was also positively correlated with IADL (*r* = 0.25).

Multicollinearity diagnostics were run before the regression analysis. The tolerance levels (0.64–0.92) and Variance Inflation Factor (VIF; 1.09–1.55) scores were all in the range of acceptability. Furthermore, the Durbin–Watson value (2.09) confirmed a lack of autocorrelation and, therefore, independence of the errors was ensured.

### 3.3. Factors Influencing IADL

Table 3 summarizes the findings from the hierarchical linear regression analysis performed to identify potential factors of IADL among elderly people living independently in the community. First, the comorbidity status that showed the biggest contribution in explaining the variance of functional performance was considered as a covariate. Model 1 exhibited a good fit with the data (*F* = 9.56, *p* = 0.002) and explained 26% of the variation in IADL. Second, when cognitive function was introduced, Model 2 accounted for 30% of the variance in IADL (*F* = 9.34, *p* < 0.001). Daily activities could be significantly predicted by these two factors, i.e., comorbidity and cognitive function (*B* = 0.37, *p* = 0.008; *B* = 0.11, *p* = 0.004). In step 3, digital literacy was introduced into the model to investigate whether this factor is related to IADL, after both comorbidity and cognitive function were controlled. The explanatory power of Model 3 climbed to 39%, a 9% increase in the variance explained relative to Model 2, and the model fit was still statistically significant (*F* = 6.49, *p* < 0.001). Although comorbidity status (*B* = 0.34, *p* = 0.017) was still a significant predictor of IADL, cognitive function and digital literacy failed to be statistically significant (*B* = 0.08, *p* = 0.052; *B* = 0.07, *p* = 0.370).

### 3.4. Moderating Effect of Digital Literacy

To determine whether digital literacy also plays a role in the link between cognitive function and IADL, a moderation model (Model 1) was run using Hayes’ PROCESS software [17]. After mean-centering independent and control variables, a statistically significant interaction term between cognitive function and digital literacy was observed (*B* = 0.03, *p* < 0.001), ultimately showing a significant moderating effect (Table 4).

**Figure 1:**
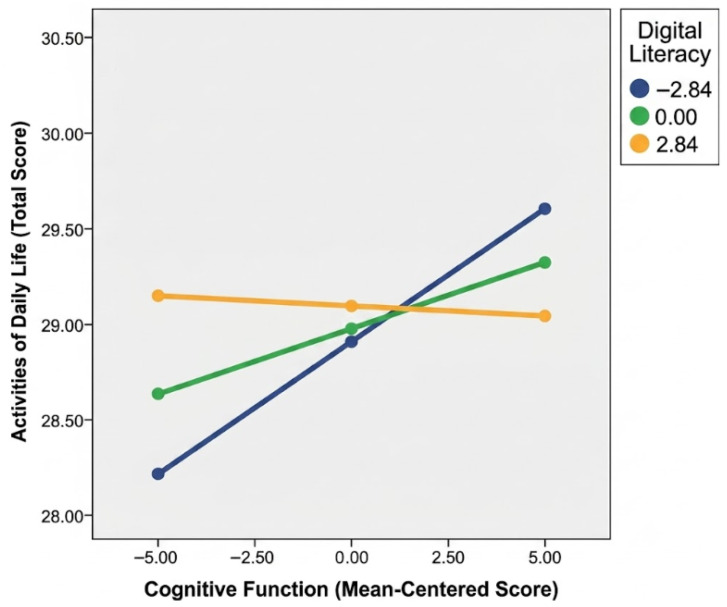
Simple slopes of the relationship between cognitive function and activities of daily life at different levels of digital literacy. *Note.* Digital literacy levels are presented as −1 SD (low), mean, and +1 SD (high). The positive association between cognitive function and activities of daily living was significant only at low levels of digital literacy.

By employing the simple slope analysis method described by Aiken & West [18], different levels of digital literacy, namely, low (−1 SD), moderate (Mean), and high (+1 SD), were considered for studying the relationship between independent and dependent variables (Figure 1). It was found that for people with low digital literacy, higher cognitive function was significantly linked to better performance in IADL (*B* = 0.12, *p* = 0.005). Conversely, the relation between cognitive function and digital literacy was not statistically significant when digital literacy was at moderate or high levels (*B* = 0.03, *p* = 0.439; *B* = −0.05, *p* = 0.399).

## 4. Discussion

The present study investigated how digital literacy might modify the effects of cognitive function on IADL in elderly community-dwelling people (75+ years). The results provide strong evidence that digital literacy can help to maintain functional independence and enhance daily life performance in a digitally connected environment. In agreement with earlier studies, cognitive function was positively related to IADL, thus further supporting the idea that cognitive impairment is the main factor leading to functional limitations [3,4]. Cognitive domains, such as memory, attention, and executive function, are indispensable for the performance of instrumental activities like medication management, financial issues, and transportation use [2]. As a result, we showed that cognitive ability is still a key element in the maintenance of independence in the elderly population.

When digital literacy and cognitive function were included in the regression model, the direct effect of cognitive function on the ability to perform IADL became non-significant. Such a finding is not surprising, as the cognitive-functional outcomes are influenced by both environmental and individual factors. In other words, digital literacy seems to be an important factor that changes the relationship between cognitive capacity and functional status. Conceptually, digital literacy may serve as a compensatory resource via digital proxies; however, since our binary tool did not measure daily behavioral integration, these mechanisms remain speculative. Future qualitative or mixed-methods studies are recommended to explore the exact pathways through which older adults utilize specific digital tools to navigate functional declines.

The main contribution of this study is the finding that digital literacy has a strong moderating influence on the cognitive–functional relationship. Our results demonstrated that changes in cognitive function significantly influenced IADL in the low-digital-literacy subgroup, whereas in the moderate- and high-literacy groups, this effect was diminished. These findings suggest that digital literacy is a protective factor that reduces the cognitive load involved in performing daily activities. Older people with better digital literacy are probably the ones who use different types of digital assistance (e.g., alert notifications, navigation systems, health-related applications) to help them with their daily instrumental activities, and thus, they are able to maintain their independence even if their cognitive abilities decline.

The results of this study are in line with earlier studies showing that digital technology improves self-care, healthcare availability, and social interaction among the elderly [7,8]. In addition, as technology helps elderly people to keep their functional independence in the community, it is considered one of the most important factors of successful aging through technology adoption and usage [9]. In this sense, digital literacy is not only a technical skill but also a major ability that promotes functional resilience and independence in older age.

The results of the present investigation have important implications for clinical and practical work. In the past, interventions for improving IADL have primarily been addressed through physical rehabilitation or cognitive training. This study supports the idea that increasing digital literacy results in living independently, i.e., digital literacy programs can even lessen the rate of functional decline in older adults with cognitive impairment. Geriatric professionals need to consider digital literacy as part of routine assessments, and they could advise on the use of digital health tools for everyday management. Moreover, the lack of digital access may make health disparities worse in an environment where healthcare is becoming more and more dependent on digital tools [10]. Educational programs along with digital environments designed for older people could not only help delay functional losses but also reduce healthcare costs. Baseline analysis showed that the low digital literacy subgroup was predominantly older, female, and low-income, highlighting that digital exclusion intersects with social vulnerability. Consequently, tailored digital literacy interventions must be designed for lower socio-economic backgrounds, providing free training and simplified interfaces to support healthy aging in place.

Despite these contributions, we should also recognize methodological flaws in our research. First, the cross-sectional design does not allow us to establish causal-effect relationships between cognitive function, digital literacy, and IADL. We need future longitudinal studies or intervention trials to clarify causal relationships. Reverse causation remains a distinct possibility. For example, older adults who better maintain independent functional capabilities (ADLs) may have more physical and motivational resources to adopt and use digital tools. Moreover, this study is vulnerable to unmeasured confusion, as it does not take into account detailed data on access to basic technologies (e.g., smartphone ownership, internet availability), intrinsic motivation for technology use, or financial and structural barriers. Second, the study sample was recruited from a single geographic area and was heavily skewed toward female participants and individuals with low economic status. Older studies suggest that low-income single women are often more vulnerable to both cognitive decline and digital exclusion [19,20,21]. Therefore, care must be taken when generalizing these findings to all elderly subjects. Third, digital literacy was measured with a self-reported yes/no scale, and therefore, such a measure might not sufficiently represent all aspects of the construct. Fourth, due to procedural constraints aimed at preventing cognitive fatigue in participants aged 75 and older, key confounding variables such as depression, social support, and prior digital device experience were not measured. Although we adjusted for comorbidity status (including physical limitations like musculoskeletal conditions), future studies should comprehensively control for these psychosocial and experiential covariates to isolate the independent effect of digital literacy.

## 5. Conclusions

Our study found that digital literacy significantly moderates the association between cognitive function and IADL among community-dwelling older adults. These findings suggest that digital literacy may function as a compensatory resource that attenuates the link between cognitive decline and functional limitations. However, the degree to which these cognitive abilities affect daily living activities depends on the digital literacy level. Specifically, being skilled in the use of digital technologies is considered a compensatory resource that lessens the effect of cognitive impairment on functional status. These findings highlight the necessity of integrating digital literacy into strategies aimed at facilitating aging in the home and sustaining functional longevity.

From clinical and policy viewpoints, improving digital literacy in the elderly population has been recognized as an effective and scalable solution to increase health-related results and to limit functional deterioration in the context of a society that is becoming more and more digital. Future research should continue to explore the cause-and-effect relationship and also measure the effectiveness of digital literacy interventions as a means to achieve sustainable aging and to preserve the ability of elderly people to function independently.

## Figures and Tables

**Table 1:** Difference in activities of daily life according to general characteristics (*n* = 147).

Characteristics	Categories	n (%) /M ± SD	Activities of Daily Life	
			M ± SD	t or F (*p*)
Age	Years	79 ± 4.33		
Gender	Male	30 (20.4)	28.63 ± 3.84	0.95 (0.350)
	Female	117 (79.6)	29.31 ± 1.72	
Highest education level	None	27 (18.4)	28.67 ± 2.39	0.69 (0.599)
	Elementary school	49 (33.3)	29.24 ± 1.79	
	Middle school	33 (22.4)	29.03 ± 3.42	
	High school	27 (18.4)	29.48 ± 1.87	
	Above college	11 (7.5)	29.82 ± 0.60	
Marital status	Married	64 (43.5)	29.16 ± 2.03	0.06 (0.941)
	Widowed	77 (52.4)	29.17 ± 2.61	
	Divorced	6 (4.1)	29.50 ± 1.22	
Income	Lower (<1.5 million KRW)	126 (85.7)	29.07 ± 2.46	1.09 (0.339)
	Moderate (1.5–3.0 million KRW)	15 (10.2)	30.0	
	Higher (>3.0 million KRW)	6 (4.1)	29.33 ± 1.62	
Comorbidity	None ^a^	9 (6.1)	29.44 ± 1.13	6.23 (<0.001)
	Hypertension ^b^	43 (29.3)	29.49 ± 1.65	(a, b, c, d, e, f > g)
	Cardiovascular disease ^c^	40 (27.2)	29.43 ± 1.52	
	Diabetes ^d^	26 (17.7)	29.23 ± 2.08	
	Peripheral vascular disease ^e^	24 (16.3)	28.88 ± 2.11	
	Cerebral vascular disease ^f^	2 (1.4)	30	
	Musculoskeletal disease ^g^	3 (2.0)	22.00 ± 9.85	

Note. M = Mean; SD = Standard Deviation. Statistical analyses were performed using independent *t*-tests or one-way ANOVA. Post hoc comparisons were conducted using the Scheffé test. Group ‘g’ (Musculoskeletal disease) scored significantly lower in IADL compared to all other medical categories.

**Table 2:** Correlation between the study variables (n = 147).

Variables	Cognitive Function	Digital Literacy	Daily Life Activities
	r (*p*)	r (*p*)	r (*p*)
Cognitive function	1		
Digital literacy	0.56 * (<0.001)	1	
Daily life activities	0.26 * (<0.001)	0.25 * (<0.001)	1

* *p* < 0.05.

**Table 3:** Factors influencing activities of daily life (n = 147).

Model	Variables	B	SE	B	t (*p*)	Adj.R^2^	F (*p*)
1	Comorbidity	0.43	0.14	0.51	3.10 (0.002)	0.26	9.56 (0.002) *
2	Comorbidity	0.37	0.14	0.22	2.71 (0.008)	0.30	9.34 (<0.001) *
	Cognitive function	0.11	0.04	0.23	2.94 (0.004)		
3	Comorbidity	0.34	0.14	0.20	2.41 (0.017)	0.39	6.49 (<0.001) *
	Cognitive function	0.08	0.05	0.19	1.96 (0.052)		
	Digital literacy	0.07	0.08	0.09	0.90 (0.370)		

* *p* < 0.05.

**Table 4:** Moderating effect of digital literacy on the influence of cognitive function on activities of daily life (*N* = 147).

Variables	B	SE	t (*p*)	LLCI	ULCI
Interaction effect					
Cognitive function × Digital literacy	−0.03	0.01	−2.54 (<0.001)	−0.05	−0.01
Conditional effects					
Level of literacy					
−1 SD	0.12	0.04	2.80 (0.005)	0.04	0.20
Mean	0.03	0.04	0.78 (0.439)	−0.05	0.12
+1 SD	−0.05	0.06	−0.84 (0.399)	−0.18	0.07

## Data Availability

Data are available from the corresponding author upon reasonable request.

## References

[1] Wiles J.L., Leibing A., Guberman N., Reeve J., Allen R.E.S. (2012). The meaning of aging in place to older people. Gerontologist.

[2] Suyasa I.G.P.D., Agustini N.L.P.I.B., Sutini N.K. (2026). Determinants of functional limitations in basic and instrumental daily activities among older adults living in rural settings: A cross-sectional study. J. Ners.

[3] Santos Henriques R.P.D., Tomas-Carus P., Filipe Marmeleira J.F. (2023). Association between neuropsychological functions and activities of daily living in people with mild cognitive impairment. Exp. Aging Res..

[4] Van Grootven B., van Achterberg T. (2022). Prediction models for functional status in community-dwelling older adults: A systematic review. BMC Geriatr..

[5] Zhang S., Zhang K., Chen Y., Wu C. (2023). Prediction models of all-cause mortality among older adults in nursing home setting: A systematic review and meta-analysis. Health Sci. Rep..

[6] Arias López M.D.P., Ong B.A., Borrat Frigola X., Fernández A.L., Hicklent R.S., Obeles A.J., Rocimo A.M., Celi L.A. (2023). Digital literacy as a new determinant of health: A scoping review. PLoS Digit. Health.

[7] Pourrazavi S., Kouzekanani K., Bazargan-Hejazi S., Shaghaghi A., Hashemiparast M., Fathifar Z., Allahverdipour H. (2020). Theory-based e-health literacy interventions in older adults: A systematic review. Arch. Public Health.

[8] Choi N.G., Choi B.Y., Marti C.N. (2025). Digital divide among homebound and semi-homebound older adults. J. Appl. Gerontol..

[9] Chimento-Díaz S., Sánchez-García P., Franco-Antonio C., Santano-Mogena E., Espino-Tato I., Cordovilla-Guardia S. (2022). Factors associated with the acceptance of new technologies for ageing in place by people over 64 years of age. Int. J. Environ. Res. Public Health.

[10] Lythreatis S., Singh S.K., El-Kassar A.N. (2022). The digital divide: A review and future research agenda. Technol. Forecast. Soc. Change.

[11] Nasreddine Z.S., Phillips N.A., Bedirian V., Charbonneau S., Whitehead V., Collin I., Cummings J.L., Chertkow H. (2005). The Montreal Cognitive Assessment, MoCA: A brief screening tool for mild cognitive impairment. J. Am. Geriatr. Soc..

[12] Kang Y., Park J., Yu K., Lee B. (2009). A reliability, validity, and normative study of the Korean-Montreal Cognitive Assessment (K-MoCA) as an instrument for screening of vascular cognitive impairment. Korean J. Clin. Psychol..

[13] Lawton M.P., Brody E.M. (1970). Assessment of older people: Self-maintaining and instrumental activities of daily living. Gerontologist.

[14] Won C.W., Rho Y.G., SunWoo D., Lee Y.S. (2002). The validity and reliability of Korean instrumental activities of daily living (K-IADL) scale. J. Korean Geriatr. Soc..

[15] Malotiya R., Setha S., Dhillon S.S., Padmanabhan J. (2023). Exploring the conceptual understanding of digital literacy: A framework for promoting digital literacy in the digital era. Humanit. Soc. Sci. Stud..

[16] Eshet-Alkalai Y. (2004). Digital literacy: A conceptual framework for survival skills in the digital era. J. Educ. Multimed. Hypermedia.

[17] Hayes A.F. (2022). Introduction to Mediation, Moderation, and Conditional Process Analysis: A Regression-Based Approach.

[18] Aiken L.S., West S.G. (1991). Multiple Regression: Testing and Interpreting Interactions.

[19] Dong Q., Liu T., Liu R., Yang H., Liu C. (2023). Effectiveness of digital health literacy interventions in older adults: A systematic review and meta-analysis. J. Med. Internet Res..

[20] Yang S., Xu W., Chen K. (2025). Digital literacy and its effects on older adults’ health: Exploring mechanisms and outcomes. Front. Public Health.

[21] Du Y., Niu Q., Tan G., Chao J., Jin S., Wang L. (2026). Digital engagement and cognitive function among older adults in China: Cross-sectional questionnaire study and moderated mediation model analysis. J. Med. Internet Res..

